# Influential factors on sexual function in infertile women with endometriosis: a path analysis

**DOI:** 10.1186/s12905-020-00941-7

**Published:** 2020-05-05

**Authors:** Samaneh Youseflu, Shahideh Jahanian Sadatmahalleh, Mahnaz Bahri Khomami, Malihe Nasiri

**Affiliations:** 1 Department of Midwifery and Reproductive Health, Faculty of Medical Sciences, Tarbiat Modares University, Ale-Ahmad Highway, Tehran, Iran; 2 Monash Centre for Health Research and Implementation, School of Public Health and Preventive Medicine, Monash University, Vic, Clayton, Australia; 3 Department of Biostatistics, Faculty of Medical Sciences, Shahid Beheshti University, Tehran, Iran

**Keywords:** Endometriosis, Sexual function, Path model, Sleep quality, HADS, Pain intensity

## Abstract

**Background:**

Endometriosis have a negative influence on women’s sexual life. The aim of the current study was to test a conceptual model considering the interrelated role of anxiety, depression, sleep quality, physical activity, BMI, stage of endometriosis, the intensity of dyspareunia and pelvic pain on sexual function (SF) in infertile women with endometriosis. Also test the mediating role of sleep quality, anxiety, and depression.

**Method:**

In the present cross-sectional study, 220 infertile women with a laparoscopically confirmed endometriosis were recruited. Data were collected using a socio-demographic checklist, Female Sexual Function Index (FSFI), Pittsburgh Sleep Quality Index (PSQI), Hospital Anxiety and Depression Scale (HADS), Visual Analog Scale (VAS).

**Results:**

We found that anxiety, depression, sleep quality, BMI, level of education, stage of endometriosis, and dyspareunia have a direct effect on women’s SF. In our study, sleep quality, anxiety, pelvic pain, and depression were the four major mediators that the higher scores lead to a decrease in the SF of endometriosis patients. The intensity of pelvic pain with an effect on sleep quality (SQ) and dyspareunia change women’s SF. The lower level of physical activity, and higher BMI with indirect effect thorough anxiety, and SQ can worsen SF. Also, a higher level of anxiety leads to poor SQ and depression. Anxiety with both direct and indirect effect impress women’s SF.

**Conclusion:**

It seems that the main risk factors for sexual dysfunction in women with endometriosis are higher rates of anxiety, depression, poor sleep quality, pelvic pain, and dyspareunia. In the care of women with endometriosis, not only laparoscopy and medical treatment should be performed but also psychotherapeutic and psychosexual help should be offered.

## Background

Endometriosis, affecting up to 10% of reproductive age women, is one of the most common gynecological diseases [1]. Pelvic pain and infertility are prevalent in 35–50% of affected *women* [2]. These women may also suffer from low self-esteem, psychological distress including stress, anxiety, and depression and poor social support [3, 4]. They may struggle with economic problems due to costs of diagnosis and treatment of chronic pelvic pain or infertility. These altogether are known risk factors for sexual dysfunction [5–9]. Most of the previous studies indicated that sexual dysfunction is higher in endometriosis women in comparison with healthy individuals [5, 8–11].

Sexual function is an essential and integral aspect of reproductive health and quality of life that is influenced by medical conditions, physical, interpersonal, psychological, and sociocultural factors [12]. A biopsychosocial approach is necessary to guide research and clinical care regarding women’s SF.

The bio-psycho-social impact of endometriosis has a negative impact on female SF [13]. Sexual dysfunction may lead to reduced quality of life (QOL), increased psychological disorder, interpersonal and marital difficulties [14, 15]. On the other hand, psychological issues play an important role in sexual dysfunction [14]. In women with major depressive disorder decreased desire, diminished arousal, and difficulty achieving orgasm are most common [16]. in addition, physical discomfort (such as dyspareunia or pelvic pain) can engender psychological problems [17, 18].

The purpose of the current study was to test a conceptual model considering the interrelated role of anxiety, depression, sleep quality, physical activity, endometriosis stage, the intensity of dyspareunia and pelvic pain on sexual dysfunction of women with endometriosis. Also test the mediating role of sleep quality, anxiety, and depression. According to the above objectives, our study proposes the following hypotheses (Hypotheses 1–5):

- **Hypothesis 1:** The intensity of chronic pain and dyspareunia of patients with endometriosis predict sexual dysfunction, psychological problems and poor SQ (the pain hypothesis).
- **Hypothesis 2:** The negative impact of endometriosis like depression and anxiety predict sexual dysfunction (the mental health hypothesis).
- **Hypothesis 3:** The stage of endometriosis found in laparoscopy predicts SF.
- **Hypothesis 4:** A higher level of education and physical activity will be associated with improved SF in women with endometriosis.
- **Hypothesis 5:** A higher BMI will be associated with sexual dysfunction, and worsens SQ.

## Methods

### Design and data collection

This study was a cross-sectional study of endometriosis women who attended the Infertility Clinic of Arash Hospital in Tehran in the period from May 2016 to February 2017.

In this period, the total number of currently infertile women (400 women) who underwent diagnostic laparoscopy was selected. Based on laparoscopic findings, we exclude women with abnormalities other than endometriosis. The subjects included 220 infertile women with a laparoscopic and/or histological diagnosis of endometriosis. Endometriosis stage was scored based on the revised classification of the American Fertility Society [19].

Inclusion criteria included age range of 18–45 years, absence of the history of chronic diseases or condition resulting in sexual dysfunction (such as cardiovascular disease, diabetes, hysterectomy, premature ovarian failure etc.), not using any drugs affecting sexual response cycle, married and living with husband, and having sexual intercourse in the past 4 weeks.

### Measures

Socio-demographic and anthropometric characteristics including women’ and spouses’ age, age at marriage, BMI, educational level, income, occupational status were collected for all participants. Participants were asked to report their exercise hours/week.

#### Sexual function

SF during a four-week period was assessed by the Persian version of the Female Sexual Function Index (FSFI), which has been previously validated by Mohammadi et al. [20]. The FSFI is a 19-item questionnaire that includes six main aspects of SFs including sexual desire (two items), arousal (four items), lubrication (four items), orgasm (three items), satisfaction (three items) and pain (three items). The score for all items ranges between 0 and 5 except for items 1, 2, 15, and 16 (ranging 1–5). The sum score of each domain was multiplied in its certain factor. This factor was 0.6 for desire, 0.3 for arousal and lubrication, and 0.4 for orgasm, satisfaction, and pain. The total score was calculated by adding the six domain scores, which higher scores indicating better SF.

#### Depression and anxiety

For detecting and classifying the severity of anxiety and depression, Hospital Anxiety and Depression Scale (HADS) was used. The instrument contains 14 questions and consists of two subscales including anxiety (HADS-A) and depression (HADS-D). Each question was rated on a 4-point Likert-type scale (0 = never, 1 = seldom, 2 = sometimes & 3 = always). Total scores less than 8 indicate normal range; scores 8–10 reflect mild alterations and scores more than 11 indicate clinically significant levels of anxiety/depression. The validity and reliability of this questionnaire were confirmed by Montazeri et al. [21]*.*

#### Pelvic pain and dyspareunia

The intensity of pelvic pain and dyspareunia was measured based on the Visual Analog Scale (VAS). This scale consists of a straight line that ranges from zero (no pain) to ten (the most severe pain possible). Subjects were asked to score their pain severity on a scale from 0 to 10.

#### Sleep quality

Multiple aspects of SQ during the previous month was assessed using the valid and reliable Persian version of the Pittsburgh Sleep Quality Index (PSQI) [22]. This questionnaire contains 19 items in seven domains (sleep quality, sleep latency, sleep duration, habitual sleep efficiency, sleep disturbances, use of sleeping medication, and daytime dysfunction) on a scale from 0 to 3; so the total score of PSQI is from 0 to 21. A total score higher than 5 identifies poor SQ and scores lower than 5 show absence of sleep disorder.

All participants answered all questionnaires. Eleven questionnaires have not been analyzed due to incomplete data. The risk factors of sexual dysfunction were assessed by path analysis.

### Statistical analysis

Data analysis was performed using the SPSS Software (version 21) and LISREL software (version 8.8). Bivariate correlations and descriptive analysis were used to analyze the degree of correlation between the SF of endometriosis women with sleep quality, anxiety, depression, endometriosis stage, the intensity of pelvic pain and dyspareunia.

A path model was used to evaluate the predictive effects of independent variables on SF in women with endometriosis. Path analysis is a kind of multiple regression statistical analysis that is utilized to asses causal models by testing a specific pattern of relationship between some variables. Direct, indirect, and total effects of causal relations between variables were found by path analysis.

We used the lisrel statistical program to fit the path model, which we hypothesized. For evaluation of the model fitness, RMSEA (Root mean square error of approximation), AGFI (adjusted goodness of fit index), CFI (Confirmatory Factor Analytic), and Chi-square/df were used. RMSEA values less than 0.07, Chi-square/df lower than 3, AGFI more than 0.9, and CFI more than 0.95 are indicative of a good fitting model. T-value greater than + 2 or less than – 2 were considered statistically significant.

## Results

Table 1 describes the demographic characteristics of the subjects. The means and standard deviations (SD) of the age and partner age were 32.10(5.44) and 36.63(5.59) years, respectively. Their mean BMI were 27.44 (4.66). Endometriosis was staged as 29.2% minimal (stage I), 22% mild (stage II), 25.4% moderate (stage III) and 23.4% severe (stage IV). Less than half of participants (43.06%) had academic degrees and about 23.4% of them were employed. Their mean BMI was 27.44 (4.66).

**Table 1:** Demographic and Anthropometric Characteristics of Women with Endometriosis

Characteristic	
Age (years)^a^	32. ±5.44
Partner age^a^	36.63 ± 5.59
BMI^a^	27.44 ± 4.66
Stage of endometriosis^b^	
Stage1	61 (29.2)
Stage2	46 (22)
Stage3	53 (25.4)
Stage4	49 (23.4)
Education^b^	
Lower than university	119 (56.93)
University	90 (43.06)
Occupation^b^	
Housewife	160 (76.55)
Employed	49 (23.44)

^a^Values are given as mean ± SD, ^b^Values are given as number (%), BMI; Body Mass Index

Table 2 demonstrates the Correlation (bivariate analysis) between all variables submitted to the path model. Results showed that SF was associated with anxiety (*r* = − 0.45, *p* < 0.001), depression (*r* = − 0.39, *p* < 0.001), BMI (*r* = − 0.42, *p* < 0.001), pelvic pain (*r* = − 0.36, *p* < 0.001), dyspareunia (*r* = − 0.36, *p* < 0.001), endometriosis stage (*r* = − 0.28, *p* < 0.001), SQ (*r* = − 0.44, *p* < 0.001).

**Table 2:** Correlations between SQ, anxiety, depression, pelvic pain, dyspareunia, BMI, endometriosis stage and sexual function

	1	2	3	4	5	6	7
1. Poor Sleep quality	–	–	–	–	–	–	–
2. Anxiety	0.32***	–	–	–	–	–	–
3. Depression	0.16*	0.21**	–	–	–	–	–
4. Pelvic pain	0.21**	0.24***	0.23***	–	–	–	–
5. Dyspareunia	0.06	0.15*	0.18*	0.45***	–	–	–
6. BMI	0.30***	0.25***	0.11	0.14*	0.06	–	–
7. Stage	0.10	0.05	0.06	0.22*	0.10	0.13	–
8. FSFI	− 0.44***	− 0.45***	− 0.39***	− 0.36***	− 0.36***	− 0.42***	− 0.28***

*BMI* Body Mass Index, *FSFI* Female Sexual Function Index; Values are given as Pearson coefficient using Pearson correlation test. *P < 0.05; **P < 0.01; ***P < 0.001

**Fig. 1:**
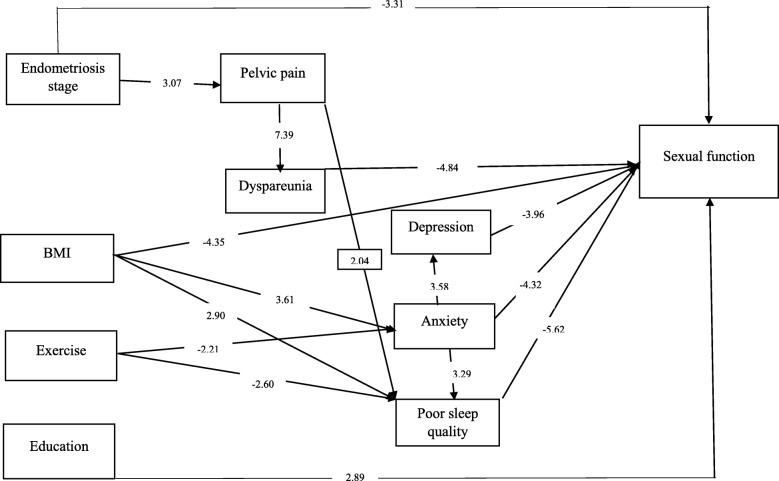
Path diagram for the predictors of sexual function in women with endometriosis

Based on the conceptual model, the predictors of SF had good fitness indices (*P*-value = 0.09; chi2 = 32.03; DF = 23; chi2/df = 1.39; RMSEA = 0.04; CFI = 0.98; AGFI = 0.93) (Table 3). Findings from the path analysis indicating the direct, indirect and total effects from the predictors on SF are reported in Table 4 and illustrated in Fig. 1.

**Table 3:** The goodness of Fit Indices for the Models

	CFI^a^	AGFI^b^	RMSEA^c^	Chi-square	df	Chi-square/df^d^	*P*-value
Path *N* = 209	0.98	0.93	0.04	32.03	23	1.39	0.09

^a^*CFI* Comparative fit index, ^b^*AGFI* Adjusted goodness fit index, ^c^*RMSEA* Root mean square error of approximation, ^d^Chi-square/df: chi-square to the degree of freedom index

**Table 4:** Path coefficients for, anxiety, depression, physical activity, BMI, sleep quality, stage of endometriosis, the intensity of pelvic pain, dyspareunia and sexual function of women with endometriosis

Predictors		Direct effect	Indirect effect	Total effect	T-value
FSFI	BMI	−0.22	−0.10	− 0.32	−6.36
	Depression	−0.20	–	−0.20	−3.96
	Physical activity	–	0.31	0.31	3.14
	Dyspareunia	−0.23	–	−0.23	−4.84
	Pelvic pain	–	−0.19	−0.19	−4.44
	anxiety	−0.23	−0.11	− 0.34	−6.16
	Poor sleep quality	−0.29	–	−0.29	−5.62
	Stage	−0.16	−0.10	− 0.26	−3.86
	Education	0.14	–	0.14	2.89
Anxiety	BMI	0.24	–	0.24	3.61
	Physical activity	−0.15	–	−0.15	−2.21
Depression	Exercise	–	−0.18	−0.18	−1.88
	BMI	–	−0.04	0.04	2.54
	Anxiety	0.24	–	0.24	3.58
Poor sleep quality	Exercise	−0.17	−0.14	−0.31	−3.06
	BMI	0.19	0.04	0.23	3.72
	Stage	–	0.09	0.09	1.70
	Pelvic pain	0.13	–	0.13	2.04
	Anxiety	0.22	–	0.22	3.29
Pelvic pain	Stage	0.21	–	0.21	3.07
Dyspareunia	Stage	–	0.25	0.25	2.83
	Pelvic pain	0.46	–	0.46	7.39

FSFI: Female Sexual Function Index, BMI: Body Mass Index

We found that anxiety (β = − 0.22), depression (β = − 0.20), SQ (β = − 0.29), BMI (β = − 0.22), level of education (β = 0.14), stage of endometriosis (β = − 0. 16), and dyspareunia (β = − 0.23) have a direct effect on women’s SF. In our study, sleep quality, anxiety, pelvic pain, and depression were the four major mediators that the higher scores lead to a decrease in the SF of endometriosis patients. The intensity of pelvic pain with effect on SQ (β = 0.13), and dyspareunia (β = 0.46) change women’s SF. The lower level of physical activity, and higher BMI with indirect effect thorough anxiety, and SQ can worsen SF. Also, a higher level of anxiety leads to poor SQ (β = 0.22) and depression (β = 0.24). Anxiety with both direct (β = − 0.23) and indirect effect (β = − 0.11) impress women’s SF.

## Discussion

The study uses a so-called pathway analysis in which all possible factors are put in to find out that indeed all factors contribute to different functions assessed in the FSFI. Recently, several studies were conducted to explore the factors affecting the SF of women with endometriosis [6, 10, 11, 23]. There is controversy regarding the strength of the relationship between physical and psychological variables and women’s SF due to uncontrolled interrelationships with various effects of modifiers, mediators or confounding variables on SF of endometriosis women.

To our knowledge, this survey is the first research that simultaneously evaluated the hypothesis that physical and psychological consequences of endometriosis are related to SF of women.

The results of the present study indicated that sleep quality, the intensity of pelvic pain and dyspareunia, physical activity, stage of endometriosis, anxiety, and depression were significant impacting SF in women with endometriosis.

In women with endometriosis, deep dyspareunia is a common symptom. Dyspareunia can be triggered by mechanical pressure on endometriotic lesion during intercourse or by traction of scarred and anelastic endometriotic lesion. Pain during intercourse can increase the risk of another type of sexual dysfunction (such as disorders in desire, lubrication, arousal, and orgasm) [24]. In Evangelista et al.’s study, endometriosis women had more dyspareunia than healthy women. However, there was no significant relationship between total FSFI score in women with and without endometriosis [25]. While in Tripoli et al. study [9], chronic pelvic pain related to endometriosis or other gynecologic disorders led to a reduction in female SF (reduces the frequency of vaginal intercourse, orgasms, and sexual satisfaction) and QOL. Mauro Cozzolino et al. reported that there was no significant difference between mean FSFI scores of the two groups (with and without pain), only endometriotic lesions at the rectovaginal site, was associated with more impaired sexual activity and SF [23]. The result from the Shum study demonstrated that worse sexual quality of life was associated with severe deep dyspareunia, severe superficial dyspareunia, increased depression, higher pain catastrophizing, bladder pain syndrome, and heterosexual orientation [5]. In one qualitative study, endometriosis-related dyspareunia had a negative impact on women’s lives (such as avoiding sexual activity, reduced self-esteem, and quality of couple relationships) [26].

In these patients, dyspareunia is not the only determinative factor of sexual health. Many factors such as chronic pelvic pain, advanced endometriosis stage and the presence of psychological and physical comorbidities, personality traits and women’s expectations affect women’s SF [15]. The result of the present study showed that anxiety and depression have more impact on the SF of women with endometriosis; a higher score indicates a more negative effects on women’s SF. Our results are inconsistent with the finding of some similar studies conducted in other countries [27–31]. Graaff et al.’s study demonstrates, dyspareunia and depressive symptoms in women with endometriosis have a negative impact on SF, however, their male partner’s SF is not affected by women’s disease [27]. The results of Finn et al.’s study on the level of sexual satisfaction in men and women with chronic pain demonstrates when psychological variables such as anxiety on sexual satisfaction are considered, variables of physical such as pain accounts for very low additional variance [32].

Our findings further showed that physical activity was the variable that significantly affected the SF of women with endometriosis. In women, increased endocrine factors (such as estrogen, oxytocin, testosterone, and cortisol) and alpha-amylase level (as a marker of sympathetic nervous system activity) following exercise can improve physiological sexual arousal [33]. In the other hand chronic exercise is related to the improvement of body image and psychological factors that increase sexual well-being [33, 34].

To our knowledge, the influencing factors on sexual dysfunction in Iranian women with endometriosis have not been assessed before. The combination of the evaluated domains (anxiety, sleep, depression, pelvic pain, endometriosis stage) is the major strength of this study as endometriosis is already well known to show interactions with each domain separately. Other strengths of the current study include confirmed diagnosis through laparoscopy, a developed conceptual model (path diagram in Fig. 1), and the use of validated questionnaires (eg, FSFI, HADS, VAS scale, PSQI, etc.).

Despite the strengths of this study, the results have some limitations. One of the limitations of this study is that we did not consider the impact of other variables which can affect SF (such as hormonal level, love, Intimacy, partner violence, and intrapersonal relationship, etc.). It has been suggested that future studies consider these issues. The selection of patients who are all recruited from an infertility unit is another limitation of this study. Suffer from infertility itself is a risk factor of sexual dysfunction with complex dynamics depending on the duration of infertility, cause of infertility and chosen treatment with the invasiveness of the procedure, financial burden, etc.

In many Asian countries, sexual issues are overlooked, ignored and considered taboo [35, 36]. Regarding the religion and culture of Iranian women, talk about sexual behaviors are considered as stigma and embarrassment, thus there might be a social desirability bias among the subjects [37, 38]. Also, we did not use a validated tool for the evaluation of physical activity. Our results only apply to married women as we excluded unmarried women.

## Conclusion

Regarding the influence of endometriosis on women’s physical and mental health, it is not unlikely that the highest impact of endometriosis on sexual dysfunction is exerted by anxiety, depression, sleep quality, pelvic pain, and dyspareunia. Also, results suggest that despite the potentially impairing impacts of pelvic pain and dyspareunia, other factors such as endometriosis stage, educational status, physical activity, and BMI may affect SF. In the care of women with endometriosis, not only laparoscopy and medical treatment should be performed but also psychotherapeutic and psychosexual help should be offered.

## Data Availability

The data sets used and analyzed during the current study are available from the corresponding author on reasonable request.

## Abbreviations

FSFI: Female sexual function index
SF: Sexual function
PSQI: Pittsburgh sleep quality index
HADS: Hospital anxiety and depression scale
VAS: Visual analog scale
SQ: Sleep quality
QOL: Quality of life
BMI: Body mass index
RMSEA: Root mean square error of approximation
AGFI: Adjusted goodness of fit index
CFI: Confirmatory factor analytic
DF: Degree of freedom
